# Methanogenic archaea and sulfate reducing bacteria co-cultured on acetate: teamwork or coexistence?

**DOI:** 10.3389/fmicb.2015.00492

**Published:** 2015-05-27

**Authors:** Derya Ozuolmez, Hyunsoo Na, Mark A. Lever, Kasper U. Kjeldsen, Bo B. Jørgensen, Caroline M. Plugge

**Affiliations:** ^1^Agrotechnology and Food Sciences, Laboratory of Microbiology, Wageningen UniversityWageningen, Netherlands; ^2^Division of Microbial Ecology, Department of Microbiology and Ecosystem Science, University of ViennaVienna, Austria; ^3^Department of Environmental Sciences, Institute of Biogeochemistry and Pollutant Dynamics, Eidgenössische Technische Hochschule ZurichZürich, Switzerland; ^4^Center for Geomicrobiology, Department of Bioscience, Aarhus UniversityAarhus, Denmark

**Keywords:** metabolic flexibility, microbial interactions, *Methanosaeta*, *Methanococcus*, *Desulfovibrio*, *Desulfobacter*

## Abstract

Acetate is a major product of fermentation processes and an important substrate for sulfate reducing bacteria and methanogenic archaea. Most studies on acetate catabolism by sulfate reducers and methanogens have used pure cultures. Less is known about acetate conversion by mixed pure cultures and the interactions between both groups. We tested interspecies hydrogen transfer and coexistence between marine methanogens and sulfate reducers using mixed pure cultures of two types of microorganisms. First, *Desulfovibrio vulgaris* subsp. *vulgaris* (DSM 1744), a hydrogenotrophic sulfate reducer, was cocultured together with the obligate aceticlastic methanogen *Methanosaeta concilii* using acetate as carbon and energy source. Next, *Methanococcus maripaludis* S2, an obligate H_2_- and formate-utilizing methanogen, was used as a partner organism to *M. concilii* in the presence of acetate. Finally, we performed a coexistence experiment between *M. concilii* and an acetotrophic sulfate reducer *Desulfobacter latus* AcSR2. Our results showed that *D. vulgaris* was able to reduce sulfate and grow from hydrogen leaked by *M. concilii*. In the other coculture, *M. maripaludis* was sustained by hydrogen leaked by *M. concilii* as revealed by qPCR. The growth of the two aceticlastic microbes indicated co-existence rather than competition. Altogether, our results indicate that H_2_ leaking from *M. concilii* could be used by efficient H_2_-scavengers. This metabolic trait, revealed from coculture studies, brings new insight to the metabolic flexibility of methanogens and sulfate reducers residing in marine environments in response to changing environmental conditions and community compositions. Using dedicated physiological studies we were able to unravel the occurrence of less obvious interactions between marine methanogens and sulfate-reducing bacteria.

## Introduction

Marine coastal and shelf sediments are important sites for mineralization of organic matter deposited from land and from the marine photic zones (Jørgensen, 1983). It is well established that under anoxic conditions, mineralization of complex organic matter requires cooperation between at least three trophic guilds (Schink and Stams, 2013). The first step in the degradation of organic matter is the hydrolysis of complex molecules into their oligomers or monomers. This step is followed by fermentation involving the degradation of these substrates to reduced organic compounds such as short chain fatty acids, and alcohols. In sulfate-rich sediments, sulfate-reducing bacteria (SRB) can use the products of primary fermentations and oxidize them to CO_2_. However, in sulfate-depleted methanogenic sediments, short chain fatty acids and alcohols are converted by secondary fermenters to acetate, formate, H_2_ and CO_2_, which are subsequently utilized by methanogenic archaea (MA) to produce CH_4_ (McInerney et al., 2008; Muyzer and Stams, 2008; Stams and Plugge, 2009; Schink and Stams, 2013).

Acetate is a key intermediate in marine sediments as it is one of the major end-products of fermentation and serves as a primary substrate for several terminal electron accepting processes, like sulfate reduction and methanogenesis (Sørensen et al., 1981; Jørgensen, 1982; Christensen, 1984; Parkes et al., 1989; Thamdrup et al., 2000). There are two possible processes for methanogens to produce methane from acetate. In the first process acetate is cleaved to CH_4_ and CO_2_. This process is called aceticlastic methanogenesis and it is an energy-yielding reaction under standard conditions (Table 1, reaction 2). The second process, syntrophic acetate oxidation, was first proposed by Barker (1936), but attracted attention much later by Zinder and Koch (1984). Syntrophic acetate oxidation is a two-step process. It the first step, acetate is oxidized to CO_2_ by an aceticlastic microorganism with the generation of reducing equivalents, often as hydrogen. This step is endergonic and requires a hydrogenotrophic microorganism for the consumption of produced hydrogen (Table 1, reaction 1). In the second step, hydrogenotrophic methanogens scavenge that hydrogen and the overall reaction becomes thermodynamically favorable (Table 1, the sum of reactions 1 and 5). Hydrogenotrophic sulfate reducers can also be involved in the second step and in case of SRB as the partner organism, the overall reaction is the same as if a sulfate reducer would oxidize acetate completely without a syntrophic partner (Table 1, the sum of reactions 1 and 3). It has been shown in previous studies that not only aceticlastic bacteria but also aceticlastic methanogens can carry out the first step of syntrophic acetate oxidation (Phelps et al., 1985). In a syntrophic relationship, the chemical energy is shared via interspecies hydrogen transfer, so that not only sulfate reducers but also the aceticlastic methanogens would be able to grow in the sulfate zone of marine sediments. It is noteworthy that the energy yield from syntrophic acetate oxidation to sulfate is greater than the energy yield from aceticlastic methanogenesis (Table 1, the sum of reactions 1 and 3).

**Table 1:** **Overview of reactions examined in this study**.

**Reaction number**	**Reactions**	**ΔG_r_**° (kJ mol^−1^)
1	CH_3_COO^−^ + 4H_2_O → 4 H_2_ + 2 HCO^−^_3_ + H^+^	214.70
2	CH_3_COO^−^ + H_2_O → CH_4_ + HCO^−^_3_	−14.74
3	4 H_2_ + SO^2−^_4_ + H^+^ → HS^−^ + 4 H_2_O	−262.06
4	CH_3_COO^−^ + SO^2−^_4_ → HS^−^ + 2 HCO^−^_3_	−47.36
5	HCO^−^_3_ + 4 H_2_ + H^+^ → CH_4_ + 3 H_2_O	−229.44

*1, Acetate oxidation; 2, Aceticlastic methanogenesis; 3, Hydrogenotrophic sulfate reduction; 4, Acetotrophic sulfate reduction; 5, Hydrogenotrophic methanogenesis, the sum of the reactions of 1 and 3 (reaction 4): Syntrophic acetate oxidation by an aceticlastic methanogen and a hydrogenotrophic sulfate-reducer, the sum of the reactions of 1 and 5 (reaction 2): Syntrophic acetate oxidation by an aceticlastic methanogen and a hydrogenotrophic methanogen. The calculations for standard conditions (298K, 1 atm, 1M reactants) were done with thermodynamic data from Lever (2012)*.

Interspecies H_2_ transfer has been studied using mixed pure cultures of the aceticlastic methanogen *Methanosarcina barkeri* and the hydrogenotrophic sulfate reducer *Desulfovibrio vulgaris* (Phelps et al., 1985). Phelphs and colleagues co-cultivated *M. barkeri* with *D. vulgaris* and reported that CO_2_ production from acetate increased and CH_4_ production decreased in cocultures compared to pure cultures of *M. barkeri*, demonstrating interspecies hydrogen transfer. Syntrophic acetate oxidation by aceticlastic methanogens and hydrogenotrophic sulfate reducers was demonstrated for anoxic paddy soils (Achtnich et al., 1995) but has not been demonstrated for marine sediments so far.

Acetate concentrations in pore water of marine sediments are reported to be relatively high [typically >10 μM (Finke et al., 2007a)] and they are likely not under thermodynamic limitation in marine sediments, which makes acetate conversion by methanogens thermodynamically feasible even in the sulfate zone (Finke et al., 2007b). However, almost all acetate in the sulfate zone is converted to CO_2_, not to CH_4_ (Jørgensen and Parkes, 2010), suggesting the predominance of aceticlastic sulfate reduction. Thermodynamic mechanisms to explain the biogeochemical zonation in marine sediments in the presence of acetate are unclear. Finke et al. (2007b) suggested that acetate oxidation might proceed via interspecies H_2_ transfer. According to their hypothesis, aceticlastic methanogenesis is exergonic as long as acetate concentrations stay above 0.05 μM. Many studies have shown the existence of methanogens in sulfate-rich marine sediments (Wilms et al., 2007; Beck et al., 2011; Schippers et al., 2012). *Methanosaeta* sp. have been detected in marine sediments (Mori et al., 2012), with unknown identities, and the marine “*Methanosaeta pelagica*” has been recently isolated (Mori et al., 2012). Aceticlastic methanogens, specifically *Methanosaeta* species, may be important in contributing to acetate degradation in marine sediments, in particular the tidal flat sediments, which have an abundant supply of organic matter.

In this study, we investigated interspecies hydrogen transfer between aceticlastic *Methanosaeta concilii* and two hydrogenotrophic microorganisms, either a sulfate reducer, *Desulfovibrio vulgaris*, or a methanogen, *Methanococcus maripaludis*. We hypothesized that the existence of interspecies hydrogen transfer between aceticlastic methanogens and hydrogenotrophic sulfate reducers/methanogens in marine sediments would help to understand what controls the distribution of methanogens in sediments. Additionally, we tested coexistence between *Methanosaetae concilii* and *Desulfobacter latus* on acetate under sulfidogenic conditions in mixed pure cultures.

## Materials and methods

### Strains and cultivation

*Methanosaeta concilii* strain (DSM 2139) was adapted to 2% NaCl conditions and maintained routinely on 10 mM acetate. *Desulfovibrio vulgaris* subsp. *vulgaris* (DSM 1744), *Desulfobacter latus* AcRS2 (DSM 3381) and *Methanococcus maripaludis* S2 (DSM 14266) were obtained from the German Collection of Microorganisms and Cell Cultures (DSMZ, Braunschweig, Germany) and maintained routinely on H_2_/CO_2_ (80:20%, v/v) plus 10 mM sulfate, 10 mM acetate plus 10 mM sulfate and H_2_/CO_2_(80:20%, v/v), respectively. All strains were grown in the same mineral salts medium (described below). Methanogenic archaea and sulfate-reducing bacteria were cultured routinely at 37°C and/or 30°C, respectively.

### Media and growth conditions

For the preparation of cocultures and maintaining the pure cultures, a marine, bicarbonate-buffered mineral salts medium was used. The anoxic medium contained the following components (grams/liter): KH_2_PO_4_ (0.41), Na_2_HPO^.^_4_2H_2_O (0.53), NH_4_Cl (0.3), NaCl (0.3), CaCl^.^_2_2H_2_O (0.11), MgCl^.^_2_6H_2_O (0.1), NaHCO_3_ (4), Na_2_S^.^9H_2_O (0.024), and 0.05% (w/v) yeast extract (YE) (added only to the pure and cocultures of *D. vulgaris* strain). The medium was supplemented with 1 ml/liter of acid and alkaline trace element solution (Stams et al., 1992). The medium was boiled and cooled to room temperature under an oxygen-free N_2_ flow. The medium was dispensed into 120 ml serum bottles. The bottles were sealed with butyl rubber stoppers and crimp caps and the gas headspace was replaced with 1.7 atm. N_2_/CO_2_ (80:20% v/v) and autoclaved.

Acetate from a concentrated sterile stock solution was added to the medium to a final concentration of 10 mM. Besides the substrate, vitamins (1 ml/liter) (Stams et al., 1992) were added from sterile stock solution to the medium. In order to reach the desired salt concentration (2%, w/v), sterile anoxic artificial seawater, containing (in grams/liter) NaCl (40), MgCl^.^_2_6H_2_O (10.8), KCl (0.7), CaCl^.^_2_2H_2_O (1) was added to serum bottles in same volume as the medium volume. The pH of the medium was set to 7.

### Experimental design

Microorganisms were cultivated in duplicate in 120 ml serum vials with a final volume of 50 ml. Complete medium (30 ml) was inoculated with 20% (v/v) of each microorganism to prepare cocultures. Final concentrations of acetate and sulfate in bacterial-archaeal cocultures were 10 mM, whereas archaeal-archaeal coculture contained only 10 mM acetate. The flasks were flushed with N_2_/CO_2_ immediately after inoculation of each strain to remove residual H_2_ and CH_4_, leaving 1.7 bar of N_2_/CO_2_ (80:20% v/v) as the headspace. All inoculations were done aseptically and all cocultures were incubated under static conditions in the dark. Cocultures of methanogenic archaea were incubated at 37°C while bacterial-archaeal cocultures were incubated at 30°C. Incubations lasted for 41 days for *M. concilii-D. vulgaris* and *M. concilii-M. maripaludis* cocultures and 21 days for *M. concilii-D. latus* cocultures. Gas and liquid samples were taken at different time intervals and analyzed for H_2_, CH_4_, acetate, sulfate, sulfide, dissolved inorganic carbon and biomass increase.

Pure cultures of respective microorganisms were cultivated in the presence of the required electron donor and acceptor as control. The culture conditions of pure cultures were explained in Section Strains and Cultivation. *D. vulgaris* was incubated at two different conditions in addition to its original culture condition; one was without H_2_/CO_2_ but with yeast extract addition (0.05%, w/v) and the other was without H_2_/CO_2_ but with YE (0.05%, w/v) and 10 mM acetate. These controls were made to check for the ability of the strain to grow and reduce sulfate with YE and/or acetate in the absence of H_2_/CO_2_.

### Analytical methods

CH_4_ was analyzed by gas chromatography with a Shimadzu GC-14B (Shimadzu, Kyoto, Japan) equipped with a packed column (Molsieve 13X, 60–80 mesh, 2 m length, 3 mm internal diameter) (Varian, Middelburg, The Netherlands) and a thermal conductivity detector set at 70 mA. The injection volume was 0.2 ml. The oven temperature and the injector temperatures were both 100°C. The detector temperature was 150°C. Argon was the carrier gas at a flow rate of 30 ml/min.

H_2_ was measured using a gas chromatograph equipped with pulsed discharge detector (PDD) (Trace Analytical, Bester, Amstelveen). The GC had Carboxen 1010 column, 3 m × 0.32 mm followed by a Molsieve 5A column, 25 m × 0.32 mm. The injection volume was 0.5 ml. The carrier gas was helium with a flow rate of 20 ml/min. The column oven temperature was 90°C, the injection oven temperature was 80°C and the detector temperature was 110°C. The pressure was 200 kPa and the input range was 64 nA.

Acetate from centrifuged (10,000 × *g*, 10 min) samples of the culture media was analyzed by Thermo Scientific Spectrasystem HPLC system equipped with a Varian Metacarb 67H 300 × 6.5 mm column kept at 30°C and running with 0.005 M sulfuric acid as eluent. The eluent had a flow of 0.8 ml/min. The detector was a refractive index detector.

Sulfate concentrations were analyzed by Thermo Scientific Dionex HPLC equipped with an AS22 column (Thermo Scientific Dionex, Massachusetts, USA) with eluents of 0.235 g/l NaHCO_3_ and 2.576 g/l Na_2_CO_3_ at a flow rate of 1.2 ml/min. The column temperature was 30°C and pressure was 130–160 bar.

Sulfide measurements were done using the methylene blue method (Cline, 1969). Samples were diluted 1:1 with 5% ZnAc solution directly after sampling, to precipitate all sulfide. The solution was stored at room temperature for at least 20 min in order to promote the precipitation of zinc sulfide. After color development, the concentration was measured on a MERCK Spectroquant® Multy at 670 nm. Demi-water was used as a blank.

The pH was measured using Proline B210 pH electrode.

### DIC measurements

For dissolved inorganic carbon (DIC) analysis, a 1 ml glass vial containing glass beads was filled with culture sample till the liquid became convex on top and the vial was sealed with a screw cap. The vials were stored at 4°C until analysis. Total DIC concentrations were measured as gaseous CO_2_ after acidification of the liquid using a gas chromatograph (SRI 310C GC, SRI Instruments Europe GmbH) equipped with a thermal conductivity detector (TCD).

### DNA extraction

Biomass was harvested at selected time points by sampling 1 mL of culture after homogenization by vortexing, and centrifugation at 13,000 g for 20 min. Genomic DNA was extracted from the pellet, using the PowerSoil^R^ DNA Isolation kit (MoBio), following the manufacturer's instructions.

### Quantification of 16S rRNA genes by quantitative PCR

The total number of 16S rRNA gene copies was quantified by SYBR Green assay, on the CFX96 TouchTM Real-time PCR Detection System (Bio-Rad). The primers used for amplifying bacterial 16S rRNA genes were Bac8F and Bac338Rabc (Juretschko et al., 1998; Daims et al., 1999). For Archaea, Arch806F and Arch958R were used (DeLong, 1992; Takai and Horikoshi, 2000). For the coculture of *Methanosaeta concilii* and *Methanococcus maripaludis*, *Methanosaeta*-specific primers (MS1b 585F and Sae 835R; Conklin et al., 2006) and *Methanococcales*-specific primers (MCC495F and MCC832R; Yu et al., 2005) were used.

Prior to qPCR, the primers were tested by end-point PCR (annealing temperature gradient from 56 to 65°C, 40 PCR cycles) on DNA extracts from pure cultures of the respective strains to ensure the specificity of the qPCR assays. None of the primer pairs used showed any unspecific amplification of non-target groups. All primers are shown in Supplementary Table 1.

Per PCR reaction, a total volume of 10 μL mixture contained 5 μL of iQ SYBR Green Supermix (Bio-Rad), 10 μM of each primer and 1 μL of ~5 ng/μl template DNA. The amplification program consisted of an initial activation step at 95°C for 3 min, 45 cycles of: denaturation at 95°C for 15 s, annealing at 55°C for 30 s and elongation at 72°C for 30 s, and a final extension step at 60°C for 31 s. For reactions involving *Methanosaeta*- and *Methanococcales*-specific primer sets, the annealing temperature was adjusted to 60°C. Melting curves were analyzed using the CFX ManagerTM software. The results were expressed as the number of cells per μL of sample, after calculating the number of 16S rRNA genes per genome from reference strains with completely sequenced genomes, using Genbank (http://www.ncbi.nlm.nih.gov/genbank) and the RNAmmer 1.2 Server (http://www.cbs.dtu.dk/services/RNAmmer/) (Lagesen et al., 2007). The calculated number of 16S rRNA gene copies and the corresponding reference strains were: 2 for *Methanosaeta concilii* GP6, 5 for *Desulfovibrio vulgaris* subsp. *vulgaris* Hildenborough^T^, 4 for *Desulfobacter postgatei* 2ac9, and 3 for *Methanococcus maripaludis* (strains C5, C6, C7, and S2).

### Calculation of Gibbs free energy (ΔG)

Gibbs free energies per reaction were calculated for the reactions shown in Table 1. For each reaction, the thermodynamic data for ΔG_f_°, ΔH_f_°, ΔV_f_° (Table S2) were used to calculate ΔG_r_° (standard Gibbs free energy of reaction), ΔH_r_° (standard enthalpy of reaction), ΔV_r_° (standard volume of reaction) (Table S3) by subtracting the sum of products from the sum of reactants.

ΔG values of reactions are dependent on temperature, pressure and chemical concentrations. Thus, ΔG_r_° values were corrected taking into account the temperature, pressure and concentrations of reactants and products (Wang et al., 2010).

Standard Gibbs free energies of reactions were corrected for different temperatures using the integrated Gibbs-Helmholz equation:

$$△G^{{}^{\circ}}\left(T\right)=T*\left(\frac{△G^{{}^{\circ}}}{298K}+\left(\frac{△H^{{}^{\circ}}}{T}-\frac{△H^{{}^{\circ}}}{298K}\right)\right)$$

The effect of pressure on ΔG° value was calculated using the equation:

$$△G^{{}^{\circ}}\left(P\right)=△G^{{}^{\circ}}\left(T\right)+△Vf^{{}^{\circ}}*\frac{\left(P-1\right)}{9869}$$

As last, Gibbs free energies per reaction were calculated using the measured products and reactants via the equation:

$$△\text{G}=△{\text{G}}^{{}^{\circ}}\text{+}RT\text{ lnK}$$

where Δ G° was calculated under standard conditions (Table S3), *R* is the gas constant (0.008314 kJ mol^−1^ K^−1^), and *T* is the absolute temperature (298.15K). The activity coefficient values for bicarbonate and acetate (0.532), for water and H^+^ (1), for H_2_ and CH_4_ (1.24), for sulfate (0.104) and sulfide (0.41) were taken from Millero and Schreiber (1982) and Lever (2012).

## Results

### *M. concilii* in coculture with *D. vulgaris*

**Figure 1:**
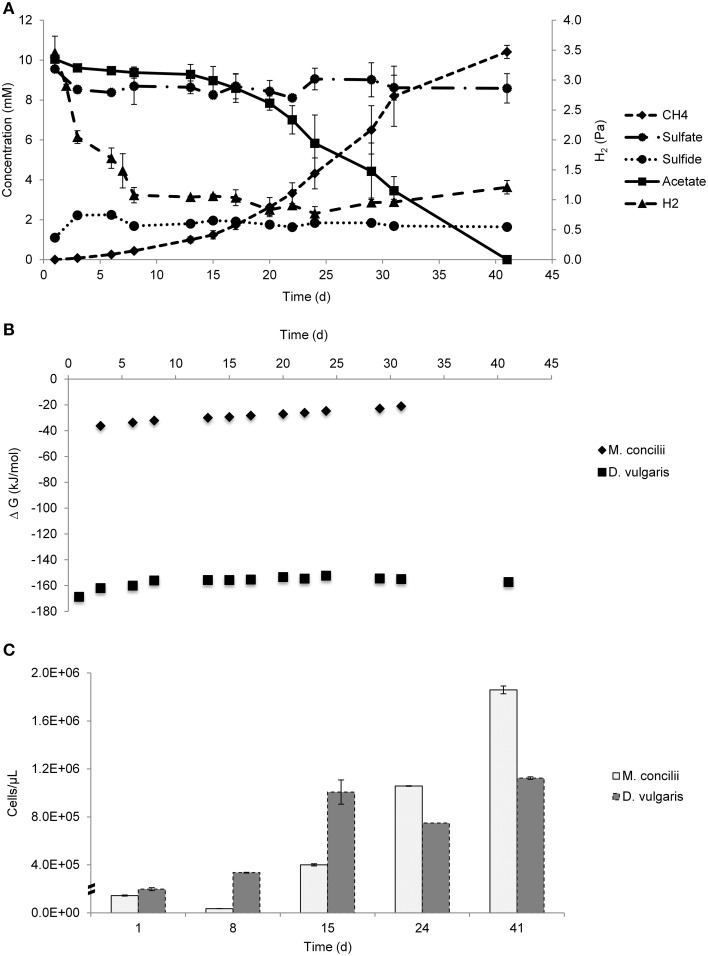
**Growth on acetate by coculture of** ***M. concilii*** **and** ***D. vulgaris*** **subsp**. ***vulgaris*****. (A)** Changes in acetate, sulfate, sulfide, methane and hydrogen. **(B)** Actual Gibbs free-energy changes for acetate degradation to sulfide and bicarbonate and methane formation from acetate. **(C)** Growth quantified by qPCR in cells/μl. All data is average of 2 replicate incubations.

Methane formation started directly and increased with time (Figure 1A). 10 mM acetate was fully converted into CH_4_. In 41 days, 0.9 mM sulfate was consumed and sulfide accumulated to a concentration of 0.8 mM. The bulk of sulfate was reduced in the first 6 days where H_2_ concentration sharply decreased. After that point, there were only slight fluctuations in sulfate concentration. The H_2_ pressure in the cocultures was 3.5 Pa when measured on day 1, presumably as a result of carryover from the *D. vulgaris* inocula. Hydrogen levels sharply decreased to 1.08 Pa in a week and then slowly dropped to 0.83 Pa until day 20. Later on, it slowly increased and reached to 1.21 Pa by the last day of the experiment. Pure cultures of *M. concilii* had pressures of 1.03 Pa H_2_ on average throughout the incubation period (Figures S1A,B). H_2_ concentration in pure culture controls of *D. vulgaris* incubated with YE and acetate without H_2_ addition was 19.5 Pa and was 8 Pa when incubated with YE only (Figures S2A,B). The pressures dropped to 0.92 Pa in both controls by the end of 8th day and remained constant during the rest of the incubation period. H_2_ concentration in control bottles did not change any further and same concentration was observed in cocultures. Thus 0.92 Pa H_2_ (equivalent to 7.12 nM) was assumed to be the threshold H_2_ concentration for the *D. vulgaris* subsp. *vulgaris* strain.

Gibbs free-energy changes in the coculture ranged between −36.2 and −20.9 kJ/mol for the conversion of acetate into methane and bicarbonate and between −168.6 and −152.3 kJ/mol H_2_ for hydrogenotrophic sulfate reduction (Figure 1B). These results showed that both reactions were favorable throughout the experiment. The most negative Gibbs free-energy values for both reactions were obtained in the beginning of experiment where acetate and hydrogen concentrations were at the highest levels. The highest Gibbs free-energy value for hydrogenotrophic sulfate reduction was −152.3 kJ/mol, showing that the growth of *D. vulgaris* was thermodynamically feasible at the determined H_2_ concentrations.

qPCR results showed an increase in cell numbers of both organisms during the experiment (Figure 1C). The decrease in the cell numbers of *M. concilii* in the first 8 days coincided with a lag phase of acetate consumption. *D. vulgaris* cell numbers increased 2-fold in the same period with H_2_ consumption coupled to sulfate reduction. Between days 8 and 15, both *M. concilii* and *D. vulgaris* had the highest increase in their cell numbers with 11- and 3-fold increase, respectively. From day 15 until day 24, cell numbers of *M. concilii* increased 2.6-fold whereas *D. vulgaris* cell numbers decreased. In the last period of the incubation, both *M. concilii* and *D. vulgaris* showed growth with 1.8- and 1.5-fold increase in cell numbers, respectively. These results showed that *D. vulgaris* grew after consuming initial hydrogen to the threshold H_2_ value.

qPCR analysis of *D. vulgaris* pure culture controls showed growth during the experiment (Figure S3). *D. vulgaris* with YE and acetate showed the highest increase in cell numbers within the first 8 days. However, *D. vulgaris* in coculture grew to the highest cell density and showed a 5-fold increase in numbers after 15 days compared to day 1.

### *M. concilii* in coculture with *M. maripaludis*

**Figure 2:**
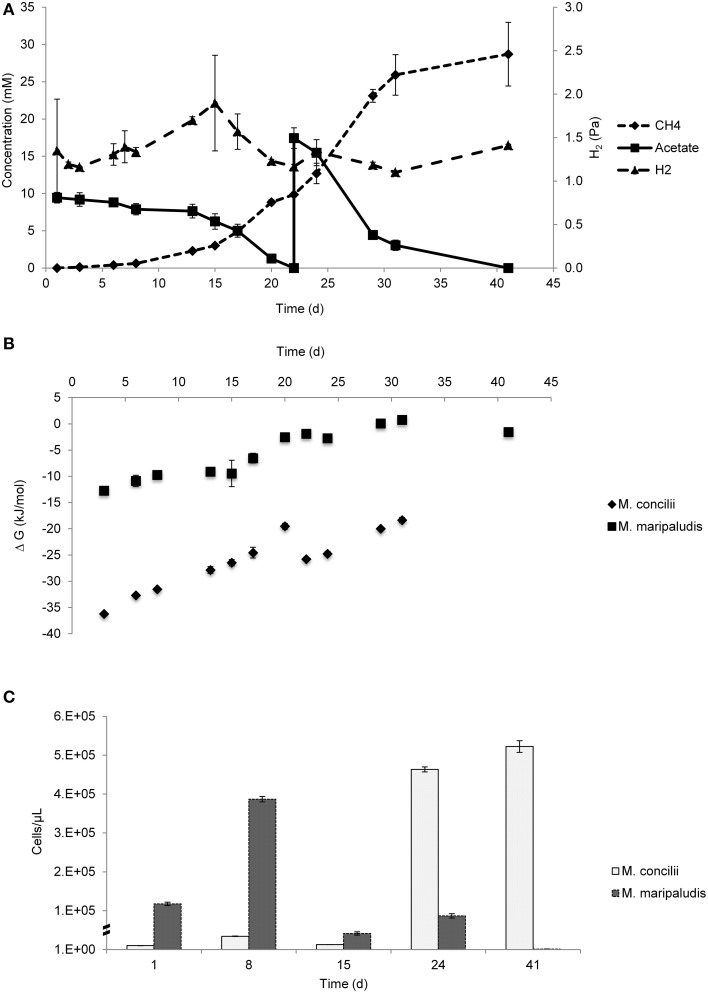
**Growth on acetate by coculture of** ***M. concilii*** **and** ***M. maripaludis*****. (A)** Changes in acetate, methane and hydrogen. **(B)** Actual Gibbs free-energy changes for acetate degradation to methane formation from acetate. **(C)** Growth quantified by qPCR in cells/μl expressed. All data is average of 2 replicate incubations.

Acetate conversion started upon the start of the experiment. CH_4_ was produced from acetate and increased rapidly after 8 days of incubation (Figure 2A). As the first acetate addition was depleted by day 22, a second feed of acetate was given to the coculture. During the course of the experiment, 27 mM acetate was consumed and 28 mM CH_4_ produced.

H_2_ level increased from 1.4 Pa to a peak concentration of 1.9 Pa during the first 15 days. This increase was concomitant to acetate consumption and CH_4_ production, which suggested H_2_ leakage from *M. concilii* cells during growth. After H_2_ reached the highest level, it was consumed by *M. maripaludis* to the lowest level which was 1.17 Pa. During the rest of the experiment, there were slight fluctuations in H_2_ level, apparent changes were not observed. In pure culture controls of *M. concilii*, average H_2_ levels were around 1.2 Pa and stayed constant throughout the experiment (Figure S1).

Gibbs free energies calculated for the conversion of acetate to methane and bicarbonate ranged between −36.2 and −18.4 kJ/mol and Gibbs free energies for hydrogenotrophic methanogenesis ranged between −12.7 and −1.5 kJ/mol H_2_ (Figure 2B). ΔG values showed that aceticlastic methanogenesis was favorable throughout the experiment. The Gibbs free energies for hydrogenotrophic methanogenesis were close to the biological energy quantum value.

According to the qPCR results, both organisms showed growth during the course of the study (Figure 2C). As a result of acetate consumption starting in the beginning of the experiment, cell numbers of *M. concilii* increased 3-fold until 8 days. Similarly, *M. maripaludis* cell numbers increased 3-fold in the first week. A decline was detected in both *M. concilii* and *M. maripaludis* cell numbers between days 8 and 15, followed by an increase simultaneous to the consumption of acetate and hydrogen. Between days 15 and 24, *M. concilii* and *M. maripaludis* cell numbers increased 36- and 2-fold, respectively. After day 24, only 1-fold increase detected in *M. concilii* cell numbers whereas a decline in *M. maripaludis* cell numbers was observed.

### *M. concilii* in coculture with *D. latus*

**Figure 3:**
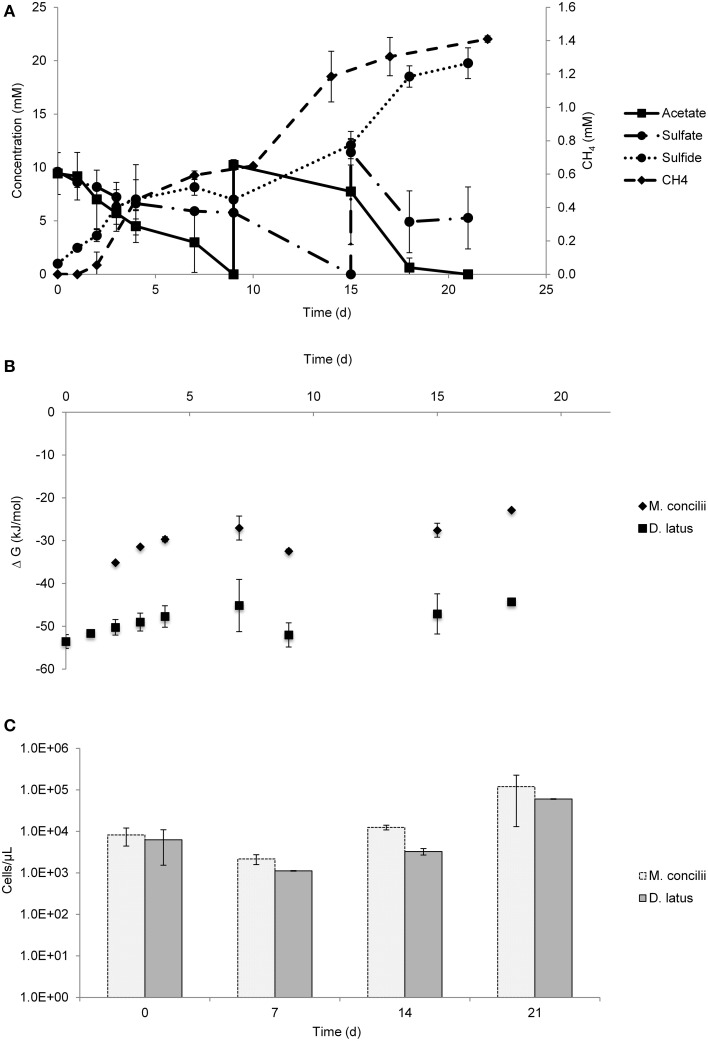
**Growth on acetate by coculture of** ***M. concilii*** **and** ***D. latus*****. (A)** Changes in acetate, sulfate, sulfide, and methane. **(B)** Actual Gibbs free-energy changes for acetate degradation to sulfide and bicarbonate and methane formation from acetate. **(C)** Growth quantified by qPCR in cells/μl. All data is average of 2 replicate incubations

Acetate conversion coupled to sulfate reduction started by the initiation of the experiment while CH_4_ production from acetate conversion was observed after a 2 day lag period (Figure 3A). Both *M. concilii* and *D. latus* contributed to acetate conversion during the experiment. *D. latus* reduced 16 mM sulfate by the oxidation of acetate, whereas *M. concilii* contributed to the acetate oxidation by producing 1.4 mM CH_4_ on average in 21 days.

Under these conditions, Gibbs free energies ranged between −44 and −54 kJ/mol for the conversion of acetate into sulfide and bicarbonate and Gibbs free energies for acetate-driven methanogenesis ranged between -23 and -35 kJ/mol (Figure 3B). ΔG values showed that both reactions were favorable during the course of the experiment.

qPCR results indicate an increase in cell numbers of both organisms during the experiment (Figure 3C). Between day 7 and 14, both *M. concilii* and *D. latus* increased their cell numbers 3.7- and 2.4-fold, respectively. The highest cell increase was observed in the last week of the experiment. Increase in cell numbers of *M. concilii* was 36-fold whereas cell numbers of *D. latus* increased 14.6-fold.

In an additional experiment where we used the same coculture combination, CH_4_ production started after few days of incubation when sulfate reduction was already ongoing (Figure S4). This coculture yielded 0.7 mM CH_4_ until all sulfate was reduced by *D. latus*, after which *M. concilii* consumed the rest of the acetate coupled to CH_4_ formation. After 53 days, 6 mM acetate was consumed by *M. concilii* stoichiometrically, which was much slower than *D. latus* (37 days).

## Discussion

In this study, we tested interspecies hydrogen transfer in two different coculture combinations. We cocultured an obligate aceticlastic methanogen, *Methanosaeta concilii* together with a hydrogenotrophic sulfate reducer, *Desulfovibrio vulgaris* or a hydrogenotrophic methanogen *Methanococcus maripaludis*. We aimed to investigate whether hydrogen leakage from *Methanosaeta* is possible under conditions where the hydrogen is efficiently scavenged by hydrogenotrophic sulfate reducers or methanogens and whether such a hydrogen leakage enables the growth of the consuming organisms. Additionally, we tested coexistence between *Methanosaetae concilii* and *Desulfobacter latus* on acetate under sulfidogenic conditions in mixed pure cultures.

### *M. concilii* in coculture with *D. vulgaris* or *M. maripaludis*

In the cocultures of *M. concilii* and *D. vulgaris*, acetate was converted into CH_4_ and CO_2_ in 1:1 stoichiometry during the incubation period. In case of syntrophic acetate oxidation by an aceticlastic methanogen and a hydrogenotrophic sulfate reducer couple, the expected overall reaction is exactly the same as if the sulfate reducer oxidized acetate completely without a syntrophic partner (Table 1, reaction 4). Taking this into account, our data on the stoichiometry of the reaction do not point directly toward such a relationship.

Sulfate reduction occurred especially in the beginning of the experiment coupled to the oxidation of residual hydrogen from the inoculum. As a result of sulfate reduction, sulfide production occurred within the same time period. A minor discrepancy between sulfide produced and sulfate consumed may be attributed to chemical oxidation of HS^−^ to polysulfide by trace levels of oxygen.

H_2_ measurements were of critical importance in our study to evaluate whether *Methanosaeta* was leaking hydrogen in coculture with a hydrogenotrophic partner. Results showed that *D. vulgaris* could couple hydrogen consumption to sulfate reduction in the first 8 days of the experiment and brought hydrogen levels to threshold concentrations and hydrogen concentrations remained at a constant low level similar to the level observed in *M. concilii* mono cultures (Figure S1). Many H_2_ measurement studies were performed in different ecosystems and in pure cultures to determine threshold H_2_ concentrations for different terminal electron accepting reactions. (Lovley, 1985; Cord-Ruwisch et al., 1988; Lovley and Goodwin, 1988; Conrad, 1996; Hoehler et al., 1998). According to these studies, threshold H_2_ concentrations for sulfate reduction were found in range between 5 and 95 nM. Our results show an average of 7 nM hydrogen in mono- and cocultures, which was in line with these observations. Taking into account that different threshold concentrations exist for growth and substrate degradation, *D. vulgaris* could benefit from traces of H_2_ leaked by *M. concilii* and coupled this to its growth. The calculated Gibbs free energy values show that the hydrogenotrophic sulfate reduction reaction was thermodynamically feasible with the hydrogen concentrations in the cocultures throughout the study (Figure 1B). Apparently, *D. vulgaris* was extremely efficient, and needed only a very little amount of hydrogen to produce sufficient energy for growth (Figure 1C). Moreover, comparing pure culture with the coculture, hydrogen levels in *Methaosaet*a suggested that cocultivation can deviate electrons towards hydrogen production (Figure 1B, Figure S1).

Thus, this result supports our hypothesis that a minor part of the acetate was converted via the production of hydrogen.

In the other coculture combination, we used *Methanococcus maripaludis*, a methanogen that can use formate and/or H_2_/CO_2_ as carbon and energy source (Jones et al., 1983), as partner organism with *M. concilii*. In the presence of the methanogen as partner organism in syntrophic acetate oxidation, the net reaction is exactly the same as if acetate was cleaved by an aceticlastic methanogen (Table 1, reaction 2). In our study, the overall stoichiometry of the reaction, with slightly higher methane production, fits with both possibilities of acetate oxidation.

The trend in hydrogen concentration was different from that the trend in hydrogen concentration in the *M. concilii* and *D. vulgaris* coculture. The initial hydrogen concentration in the coculture was lower and an increase in hydrogen production was observed between day 3 and day 15. This increase was concomitant to acetate consumption and CH_4_ production, which suggests H_2_ leakage from *M. concilii* cells during growth. In the *M. concilii* control monoculture at 37°C, there was no evidence for H_2_ accumulation as H_2_ level remained constant around 1.2 Pa throughout the experiment (Figure S1). Therefore we speculated that *M. maripaludis* induced divergence of electrons from *M. concilii* and scavenged hydrogen leaked by *M. concilii*.

Comparing both cocultures, the H_2_ concentration in *M. concilii-M. maripaludis* coculture was higher than in *M. concilii-D. vulgaris* coculture, which can be attributed to the ability of *D. vulgaris* to reduce H_2_ concentrations to lower levels than *M. maripaludis*. Our data on threshold H_2_ concentrations determined for *M. maripaludis* (~10 nM) fit with the finding of Hoehler et al. (1998) where threshold H_2_ concentrations for methanogens were reported to be around 13 nM.

ΔG values showed that aceticlastic methanogenesis was favorable throughout the experiment. On the other hand, ΔG values for hydrogenotrophic methanogenesis were close to the minimum biological energy quantum that permit organisms to grow (Hoehler et al., 2001). We used batch cultures to demonstrate the growth of both organisms. However accumulating methane in the bottles had a negative effect on the overall Gibbs free energy. If we calculate the Gibbs free energy using 1 mM of methane, a value that is more realistic in marine sediments, the energy ranges from – 7 to – 14 kJ/mol. Likewise, it was reported that methanogen yields may be −10 to −15 kJ/mol in marine sediments (Hoehler et al., 2001; Finke et al., 2007b; Jørgensen and Parkes, 2010). The decline in *M. marilaudis* cell numbers after day 24 can be explained by the decay rates of *M. maripaludis*. It is known that hydrogenotrophic methanogens have a high decay rate when left without substrate and stabilized in iron sulfide precipitates (Stams et al., 1992).

Taken together, we can speculate that the hydrogenotrophic methanogen benefited from the hydrogen leaked during the growth of the aceticlastic methanogen. Our findings on growth trend, ΔG values and aforementioned reference studies showed the capability of *M. maripaludis* to metabolize and grow on H_2_ leaked by *M. concilii*. In this context it could be speculated that the hydrogen scavengers may act as parasites, as they benefit from the leakage of hydrogen by *Methanosaeta*.

There are several studies that demonstrated interspecies hydrogen transfer in defined cocultures (McInerney and Bryant, 1981; Phelps et al., 1985; De Bok et al., 2002). In one of those studies, mixed pure cultures of *Methanosarcina barkeri* and *Desulfovibrio vulgaris* were tested for interspecies hydrogen transfer under high sulfate conditions using methanol and acetate as carbon and energy sources (Phelps et al., 1985). It is known that *M. barkeri* can produce trace amounts of H_2_ during growth on acetate in pure culture and use some of the substrate for growth (Lovley and Ferry, 1985; Phelps et al., 1985; Valentine et al., 2000). They reported decreased CH_4_ production and doubled CO_2_ formation when acetate was oxidized in coculture. Lower hydrogen concentrations were measured in coculture compared to the pure cultures of the methanogen, meaning that *D. vulgaris* consumed hydrogen produced by *M. barkeri*. The authors claimed that *D. vulgaris* caused a decrease in methanogenesis by means of linking interspecies hydrogen transfer to sulfate reduction.

*Methanosarcina* species are known to be generalists, they have low affinity for acetate and have a minimum threshold for acetate of around 0.2–1.2 mM (Jetten et al., 1992). On the other hand, *Methanosaeta* species are specialists, they consume only acetate as carbon and energy source and their minimum threshold for acetate is 7–70 μM (Jetten et al., 1992). As acetate concentrations in the pore water of marine sediments are usually less than 20 μM (between 8 and 45 μM) (Christensen and Blackburn, 1982; Wellsbury and Parkes, 1995; Finke et al., 2007a), conditions appear to be suitable for *Methanosaeta* rather than for *Methanosarcina*. Many clones closely related to *Methanosaeta* have been detected in marine sediments (Mori et al., 2012), with unknown identities, however *Methanosaeta pelagica* has been recently isolated (Mori et al., 2012). Undoubtedly, *Methanosaeta* is one of the most recalcitrant methanogens and is difficult to enrich and isolate primarily because of slow growth. Hydrogen production from *Methanosaeta* was demonstrated for *Methanosaeta thermophila* when growing on acetate (Valentine et al., 2000), and here we reported for the first time hydrogen leakage from a mesophilic halotolerant *Methanosaeta*.

### *M. concilii* in coculture with *D. latus*

*M. concilii* and *D. latus* grew well in coculture (Figure 3). Methane production occurred even in the presence of high sulfate concentrations (7 mM). In the presence of non-limiting acetate concentrations, there was only minor competition for acetate between *M. concilii* and *D. latus*, as it was indicated by the concomitant sulfate reduction and methane production starting from the beginning of the experiment. qPCR data showed that *M. concilii* had an efficient biomass production at the end of the experiment. Additional data showed the same results, with slow, but steady production of methane after depletion of sulfate (Figure S4). Taken together, it is obvious that acetate conversion by aceticlastic methanogens in the presence of high sulfate and active aceticlastic sulfate reducers is possible. The concept of SRB and methanogen predominance in high-sulfate and low-sulfate environments, respectively, was established through the accumulation of results from a vast number of studies since 1980s (Ward and Winfrey, 1985; Widdel, 1988). Later, the coexistence of methanogens and SRB was observed in the presence of non-limiting sulfate concentrations in different environments (Dar et al., 2008). Coexistence of SRB and MA has been determined in organic-rich sediments with methane production rates accounting for <10% of the sulfate reduction rates (Crill and Martens, 1986). This provides a possible explanation for the coexistence of SRB and MA in this sulfate-rich medium as the concentration of acetate either exceeds the competition level or it is used non-competitively.

### New insights in metabolic flexibility

Interspecies hydrogen transfer has been studied in different anoxic environments (e.g., freshwater and marine sediments, flooded soil, landfills, and sewage digesters) for long time and its importance and mechanism in complete mineralization of organic matter has been well-documented (McInerney et al., 2008; Stams and Plugge, 2009). Moreover, interspecies formate transfer has been put forward as an alternative way of syntrophy and equally important for electron transfer between microorganisms (Boone et al., 1989; De Bok et al., 2002, 2004). Recent studies have described a new concept, direct interspecies electron transfer (DIET), where two *Geobacter* species form large, electrically conductive aggregates and establish electrical connections via the pili of both species to transfer electrons (Summers et al., 2010). In addition, DIET has been reported to occur in coculture of aceticlastic *Methanosaeta harundinacea* and exoelectrogen *Geobacter metallireducens*. In this coculture, *M. harundinacea* was found to convert acetate produced from ethanol metabolism and accept additional electrons *via* DIET for the reduction of carbon dioxide to methane; thus ethanol was converted to methane stoichiometrically (Rotaru et al., 2014). The authors have reported that transcript abundance of the genes for the enzymes necessary for the reduction of carbon to methane was high in the aggregates (Rotaru et al., 2014). Similar findings were reported previously in comparative genome analysis study of *Methanosarcina mazei* and *Methanosaeta thermophila* (Smith and Ingram-Smith, 2007). In this study, it was shown that the two genera use different enzymes to catalyze the first step of aceticlastic methanogenesis, but the majority of the core steps of the pathway were similar, except for the differences in electron transfer and energy conservation. Additionally, they identified the genes required for enzymes to catalyze CO_2_ reduction to CH_4_ in *Methanosaeta thermophila* genome (Smith and Ingram-Smith, 2007).

Given that *Methanosaeta* genus members are unable to use hydrogen directly to reduce CO_2_, these findings become important to exhibit different metabolic capabilities of *Methanosaeta* species to survive under hydrogen and acetate deficient conditions and thrive in methanogenic environments. In another recent study, it was found that both wild type and hydrogenase-deletion mutant of *Methanococcus maripaludis* could produce methane by uptake of cathodic electrons from a graphite electrode, which serves another model to direct electron uptake by methanogens (Lohner et al., 2014). These newly proposed properties of *Methanosaeta* and *Methanoccocus* indicate a variety of mechanisms for microbial electron uptake, and suggest that these methanogens may thrive in marine sediments in close contact with each other for the ultimate metabolism of substrates and that they are capable of responding to changes in environmental conditions. Future experiments on environments with fluctuating sulfate levels could apply individual based technologies to reveal the *in situ* metabolism of the microorganisms present.

### Conclusions

In conclusion, we show that an obligate aceticlastic methanogen, *Methanosaeta concilii*, leaked sufficient hydrogen to support the growth of a hydrogenotrophic sulfate reducer, *D. vulgaris*, or a hydrogenotrophic methanogen, *M. maripaludis*, when cultured together. The other important outcome of this study was the coexistence of the aceticlastic methanogen and an aceticlastic sulfate reducer in the presence of high sulfate concentration. These results bring more insights into the metabolic flexibility of methanogens and sulfate reducers residing in marine environments to adapt to changing environmental conditions and community.

### Conflict of interest statement

The authors declare that the research was conducted in the absence of any commercial or financial relationships that could be construed as a potential conflict of interest.
