# Supplementary material for: Methanogenic archaea and sulfate reducing bacteria co-cultured on acetate: teamwork or coexistence?
Source: Front Microbiol. 2015 May 27;6:492. doi: 10.3389/fmicb.2015.00492 (PMC4445324; doi:10.3389/fmicb.2015.00492)
Supplement: Supplementary file 1 [file DataSheet1.PDF]

Table S1 PCR primers used in the study.

Primer Name	Sequence (5' – 3')	Reference
Bac8F	AGAGTTTGATYMTGGCTCAG	Juretschko et al., 1998
Bac338Rabc	GCWGCCWCCCGTAGGWGT	Daims et al., 1999
Arch806F	ATTAGATACCCSBGTAGTCC	Takai & Horikoshi, 2000
Arch958R	YCCGGCGTTGAMTCCAATT	DeLong, 1992
MS1b 585F	CCGGCCGGATAAGTCTCTT GA	Conklin et al., 2006
Sae 835R	GACAACGGTCGCACCGTGGCC	Conklin et al., 2006
MCC495F	TAAGG GCTGG GCAAGT	Yu et al., 2005
MCC832R	CACCT AGTTC GCAGAGTTTA	Yu et al., 2005

Table S2 Thermodynamic data of aqueous educts and products under standard conditions.

**Compound**	**ΔG_f° (kJ mol^{-1})**	**ΔH_f° (kJ mol^{-1})**	**ΔV_f° (cm^3 mol^{-1})**	**Reference**
CH_3COO^-	-369.4	-486.4	40.5	Shock and Helgeson (1990)
HCO_3^-	-586.9	-692.0	24.6	Wagman et al. (1982), Shock et al. (1997)
H_2O	-237.18	-285.83	18.02	Amend and Shock 2001
H^+	0.0	0.0	0.0	Shock et al. (1997)
H_2	17.6	-4.2	25.2	Wagman et al. (1982), Shock and Helgeson (1990)
CH_4	-34.47	-87.96	37.3	Shock and Helgeson (1990)
SO_4^{2-}	-744.96	-910.21	13.88	Shock et al. (1997)
HS^-	11.97	-16.12	20.65	Shock et al. (1997)

Table S3 Standard free energy of reaction (Δ*G*r°), standard enthalpy of reaction (Δ*H*r°) and standard volume of reaction (Δ*V*r°) data of aqueous educts and products under standard conditions.

**Reactions**	**ΔGr° (kJ mol^{-1})**	**ΔHr° (kJ mol^{-1})**	**ΔVr° (mol^{-1})**
$CH_3COO^- + 4\ H_2O \rightarrow 4\ H_2 + 2\ HCO_3^- + H^+$	214.70	229.12	37.42
$CH_3COO^- + H_2O \rightarrow CH_4 + HCO_3^-$	-14.74	-7.70	3.38
$4\ H_2 + SO_4^{2-} + H^+ \rightarrow HS^- + 4\ H_2O$	-262.06	-232.59	-21.95
$CH_3COO^- + SO_4^{2-} \rightarrow HS^- + 2\ HCO_3^-$	-47.36	-3.47	15.47
$HCO_3^- + 4\ H_2 + H^+ \rightarrow CH_4 + 3\ H_2O$	-229.44	-236.82	-34.04
