# Supplementary figures and images for: Methanogenic archaea and sulfate reducing bacteria co-cultured on acetate: teamwork or coexistence?
Source: Front Microbiol. 2015 May 27;6:492. doi: 10.3389/fmicb.2015.00492 (PMC4445324; doi:10.3389/fmicb.2015.00492)

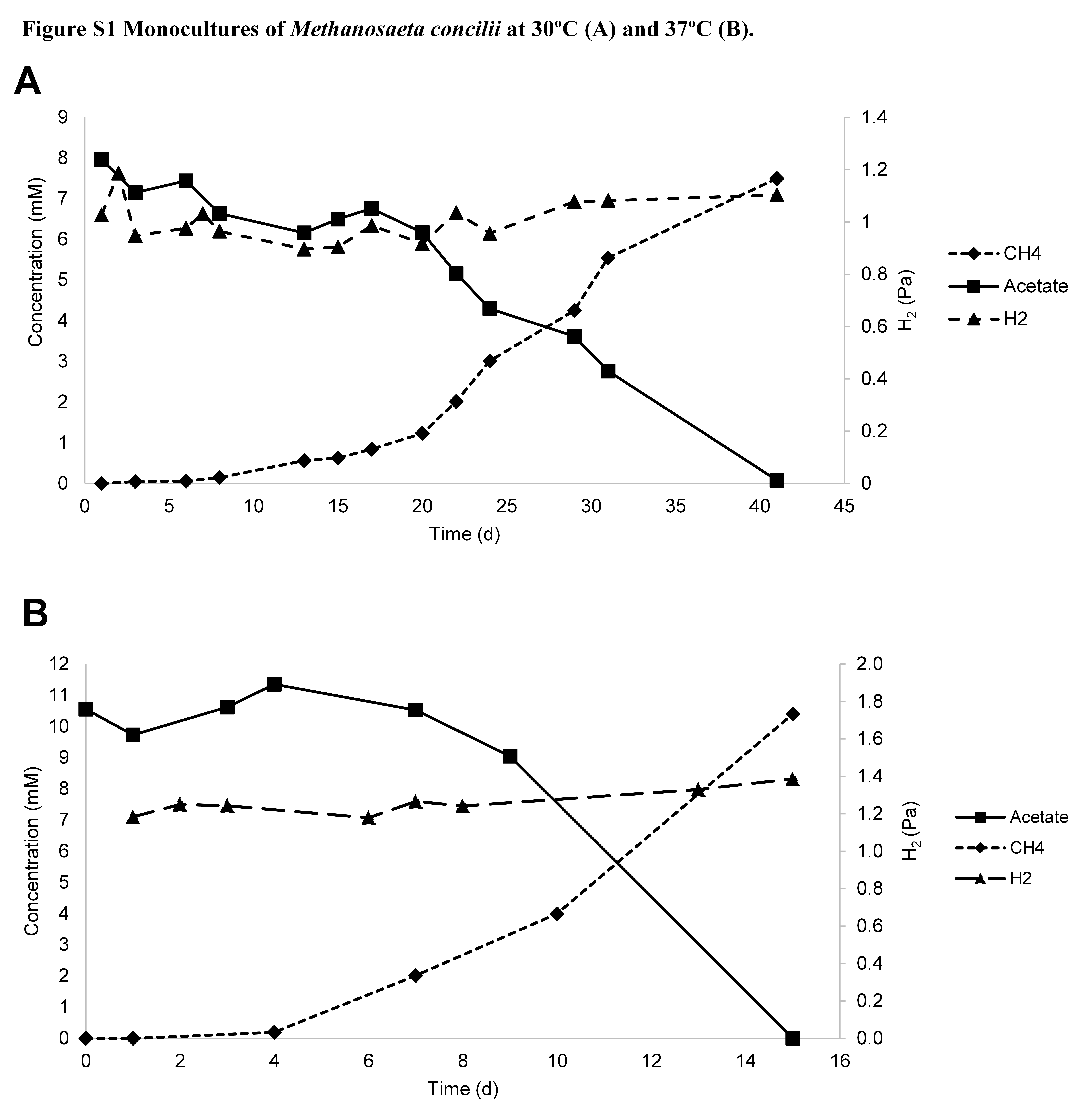

Supplement: Supplementary file 2 [file Image1.TIF]

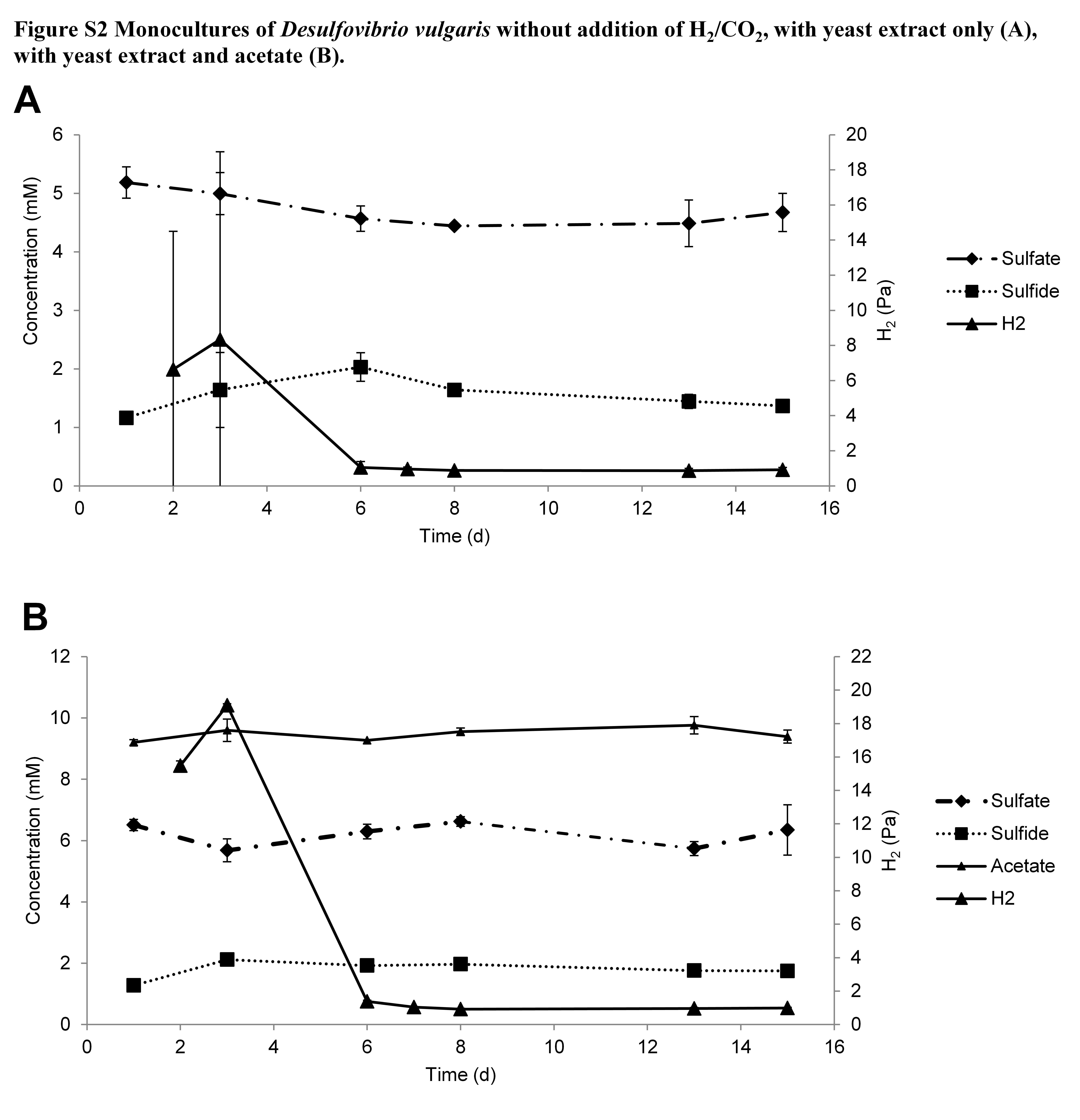

Supplement: Supplementary file 3 [file Image2.TIF]

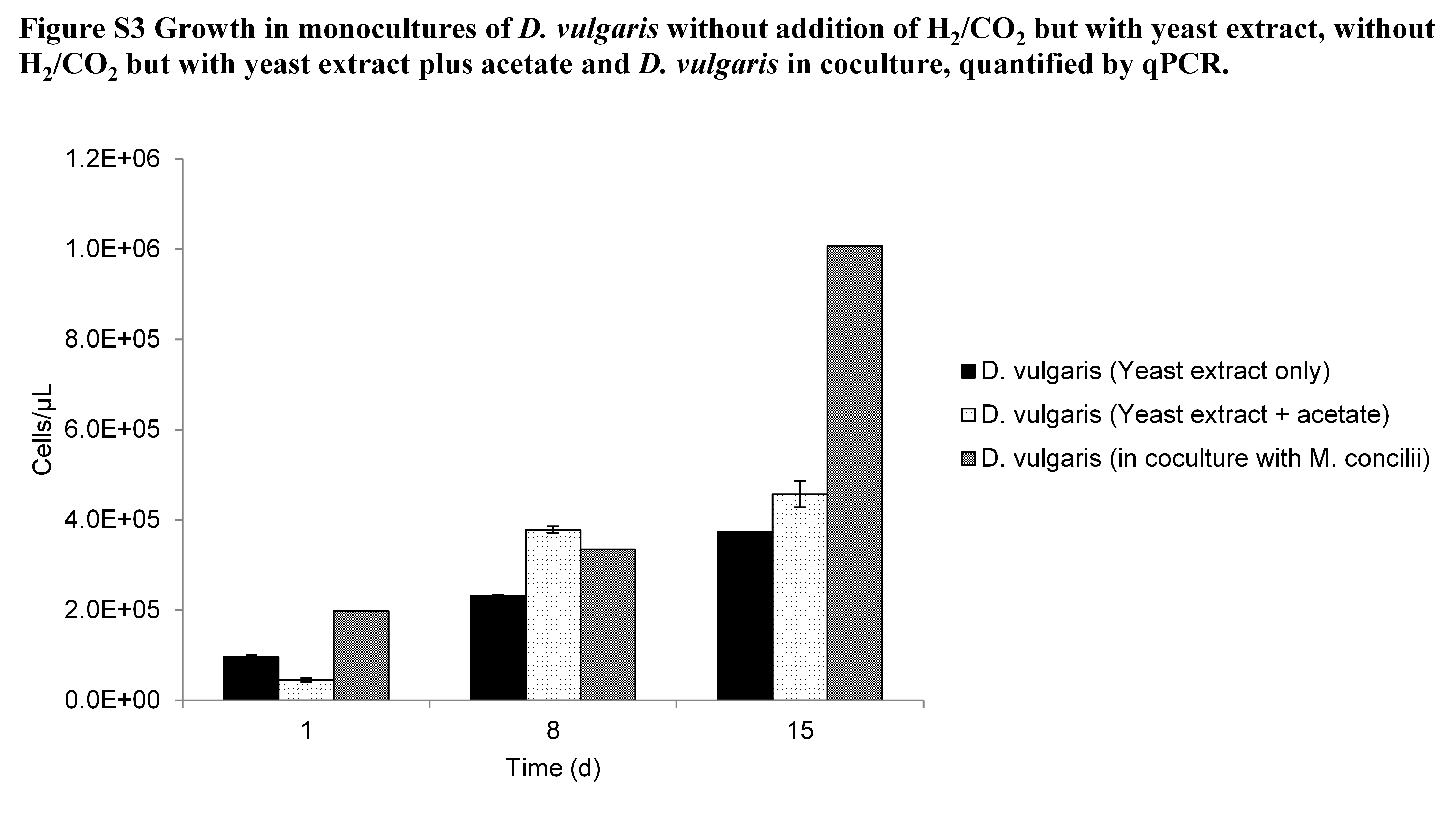

Supplement: Supplementary file 4 [file Image3.TIF]
